# Cend1, a Story with Many Tales: From Regulation of Cell Cycle Progression/Exit of Neural Stem Cells to Brain Structure and Function

**DOI:** 10.1155/2019/2054783

**Published:** 2019-05-02

**Authors:** Maria Gaitanou, Katerina Segklia, Rebecca Matsas

**Affiliations:** Laboratory of Cellular and Molecular Neurobiology-Stem Cells, Department of Neurobiology, Hellenic Pasteur Institute, Vas. Sofias Avenue, 11521 Athens, Greece

## Abstract

Neural stem/precursor cells (NPCs) generate the large variety of neuronal phenotypes comprising the adult brain. The high diversity and complexity of this organ have its origin in embryonic life, during which NPCs undergo symmetric and asymmetric divisions and then exit the cell cycle and differentiate to acquire neuronal identities. During these processes, coordinated regulation of cell cycle progression/exit and differentiation is essential for generation of the appropriate number of neurons and formation of the correct structural and functional neuronal circuits in the adult brain. Cend1 is a neuronal lineage-specific modulator involved in synchronization of cell cycle exit and differentiation of neuronal precursors. It is expressed all along the neuronal lineage, from neural stem/progenitor cells to mature neurons, and is associated with the dynamics of neuron-generating divisions. Functional studies showed that Cend1 has a critical role during neurogenesis in promoting cell cycle exit and neuronal differentiation. Mechanistically, Cend1 acts via the p53-dependent/Cyclin D1/pRb signaling pathway as well as via a p53-independent route involving a tripartite interaction with RanBPM and Dyrk1B. Upon Cend1 function, Notch1 signaling is suppressed and proneural genes such as Mash1 and Neurogenins 1/2 are induced. Due to its neurogenic activity, Cend1 is a promising candidate therapeutic gene for brain repair, while the *Cend1* minimal promoter is a valuable tool for neuron-specific gene delivery in the CNS. Mice with *Cend1* genetic ablation display increased NPC proliferation, decreased migration, and higher levels of apoptosis during development. As a result, they show in the adult brain deficits in a range of motor and nonmotor behaviors arising from irregularities in cerebellar cortex lamination and impaired Purkinje cell differentiation as well as a paucity in GABAergic interneurons of the cerebral cortex, hippocampus, and amygdala. Taken together, these studies highlight the necessity for Cend1 expression in the formation of a structurally and functionally normal brain.

## 1. Introduction

Neural stem and progenitor cells are the building blocks of the brain. In the embryo, these cells are located in proliferative zones and produce a variety of neurons and glia through tightly regulated processes that result in the generation of the diversity and complexity of the cellular phenotypes found in the adult brain [1–8]. Neural stem/precursor cells (NPCs) initially undergo symmetric proliferative divisions to expand the NPC pool and later switch to asymmetric self-renewing divisions that yield one NPC daughter cell and one daughter cell with a more committed neuronal fate. Finally, symmetric differentiative divisions take place during the late embryonic stage to produce two daughter neurons thus increasing neuronal output [5, 9, 10]. NPCs proliferate in the ventricular and subventricular zones of the developing forebrain, then exit the cell cycle, and differentiate as they migrate away from these zones. During this process, coordinated regulation of cell cycle exit and differentiation is essential for generation of the appropriate number of neurons and formation of the correct structural and functional connections of neuronal circuits. Previous studies have shown that progression of progenitor cells towards neuronal differentiation is tightly linked with cell cycle control and that the two events are coordinately regulated [9, 11–13]. Maintaining the balance between progenitor cell proliferation and neuronal differentiation is critical for the generation of the right number of neurons at the right time and place, thus ensuring proper CNS structure and function. Present evidence supports that cell cycle regulators can influence neural cell fate and differentiation, and conversely, cell fate determinants and differentiation-inducing proteins regulate the cell cycle [9, 14]. Over the years, the identification of cellular and molecular determinants that regulate and orchestrate cell cycle progression/exit and differentiation in the central nervous system (CNS) has been a subject of intense investigation with a large number of molecules implicated in the regulation of these processes. Among the different proteins that act as cell cycle regulators, Cend1 (for cell cycle exit and neuronal differentiation 1; also known as BM88) is a neuronal lineage-specific modulator that plays a role in synchronization of cell cycle exit and differentiation of neuronal progenitors in the developing nervous system. Notably, Cend1 is a neuron-specific protein which is expressed in NPCs of the embryonic forebrain and spinal cord at a time window when these cells are destined to generate neurons, while it ceases to be expressed when they give rise to glial cells [15–18]. Further, Cend1 is associated with the dynamics of neuron-generating divisions [15]. Interestingly, Cend1 marks dividing NPCs, young neurons, and terminally differentiated neuronal cells all along the neuronal lineage [15–22]. Functional studies have shown that Cend1 has a dual role during neurogenesis, promoting cell cycle exit, and neuronal differentiation. Especially, Cend1 negatively regulates proliferation via the p53/Cyclin D1/pRb signaling pathway [23] that controls the balance between cell cycle progression and exit, while its neuronal differentiation-promoting activity involves downregulation of Notch signaling and activation of proneural gene networks [16]. Moreover, tripartite functional interactions between Cend1, RanBPM, and the Mirk/Dyrk1B minibrain kinase affect Cyclin D1 levels and cell cycle progression/exit [24]. Here, we review the biochemical properties, expression profile, and functional characteristics of Cend1 and discuss its role in cell cycle progression/exit and the generation of proper brain structure and function. We also describe a recent finding that highlights its implication in Zika virus infection [25].

## 2. Biochemical Properties and Intracellular Localization of Cend1 Protein

**Figure 1:**
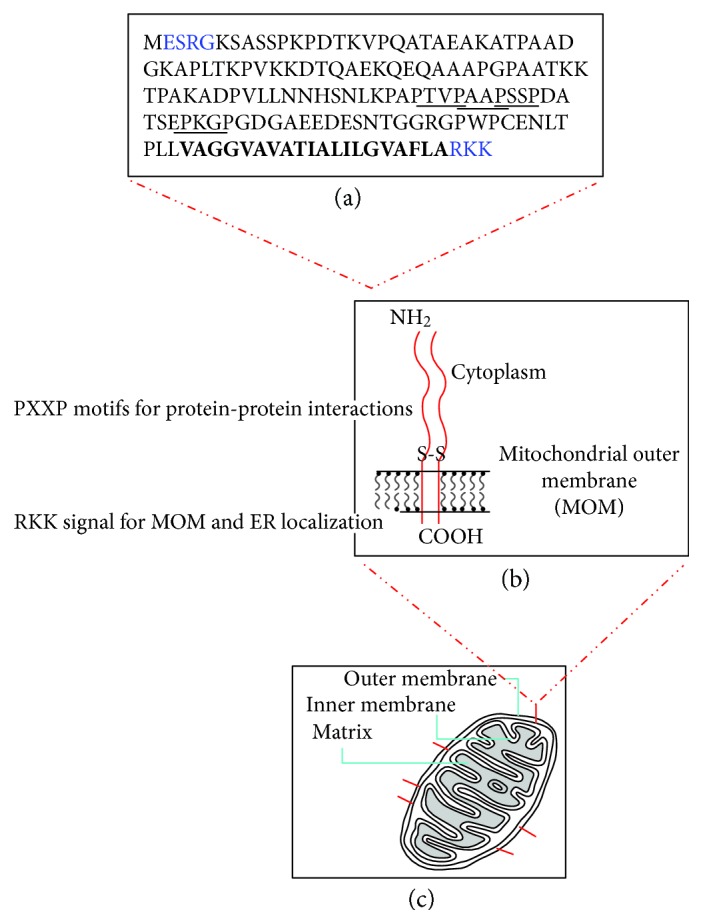
Primary structure (a) and localization of Cend1 protein in intracellular organelles. Cend1 is anchored to the lipid bilayer of MOM and ER via a 20 hydrophobic amino acid stretch shown in bold. N- and C-terminal short amino acid sequences required for proper MOM and ER targeting are in blue, and repeated PXXP motifs essential for protein-protein interactions are underlined.

Purification and biochemical characterization of Cend1 protein showed that it is an integral membrane protein apparently not glycosylated, composed of two identical 22-23 kDa polypeptide chains depending on the species tested, and linked together by disulphide bridges [17, 20, 21, 26]. Electron microscopy observations have shown that Cend1 protein is anchored to the lipid bilayer of a number of intracellular organelles, including the endoplasmic reticulum (ER), outer mitochondrial membrane (MOM), and small electron-lucent vesicles, and to a lesser extent to the plasma membrane, especially at the level of synaptic densities [20]. Cend1 consists of 149 amino acids in humans, mice, and rats, while the porcine and chick homologues consist of 140 and 130 amino acids, respectively [17, 21, 26]. Cend1 is anchored to intracellular membranes via a transmembrane domain of 20 hydrophobic amino acids, followed by a short tail of 3 positively charged residues (RKK) on the C-terminus, in such a way that the bulk of the protein faces the cytoplasm [20, 21, 26] (Gaitanou and Matsas, unpublished data) (Figure 1). The three positively charged residues (RKK) located at the C-terminus next to a hydrophobic transmembrane domain act as a MOM targeting signal (Gaitanou and Matsas, unpublished data), as has been shown for the Bcl-2 family of anti- and proapoptotic proteins [27–29]. Furthermore, short specific N- and C-terminal amino acid sequences have been shown to be essential for proper Cend1 targeting to MOM and ER (Gaitanou and Matsas, unpublished data).

Cend1 protein in all tested species contains a proline-rich signaling domain that maybe involved in protein-protein interactions [17, 21]. This proline-rich domain contains several PXXP repeats, which represent putative SH3-binding sites and have been detected in a variety of proteins comprising diverse signal-transduction pathways [30]. To date, Cend1 has been shown to interact directly with RanBPM, a promiscuous scaffolding protein of the nervous and immune systems, and Ahi1, a protein implicated in Joubert syndrome, which is a rare autosomal recessive disorder characterized by an abnormal brain structure [24, 31]. Recently, Cend1 has also been demonstrated to interact directly with NS4B, a nonstructural Zika virus protein [25]. The functional implications of these protein-protein interactions are described below.

## 3. Cend1 Is an Early Marker of the Neuronal Lineage Associated with the Dynamics of Neurogenic Divisions

### 3.1. Embryonic Neurogenesis

As previously discussed, progression of progenitors towards neuronal differentiation is tightly linked with cell cycle control and the two events are coordinately regulated. Early in the neurogenic period, the transition from a symmetric towards an asymmetric pattern of divisions marks the onset of neurogenesis [5, 32]. Conversely, during late neurogenesis, an increasing number of progenitors divide symmetrically to produce two postmitotic daughter neurons. At this time, the neurogenic potential of precursors decreases while their gliogenic potential increases [11, 33]. Numerous studies have unveiled molecules affecting neurogenic divisions, thus linking cell cycle exit with neuronal differentiation. Genetic studies on Drosophila have identified a number of genes acting to specify asymmetric divisions [34–36]. Accordingly, in vertebrates, Numb [37–41], the antiproliferative genes PC3/Tis21 [42] and pRb [43], and the transcription factor Pax6 [44, 45] seem to affect the progression from symmetric/proliferative to asymmetric/neuron-generating divisions.

Cend1 was initially identified and studied by our group as a component of mature neurons with the use of a monoclonal antibody (mab BM88) raised against Triton X-114-treated pig synaptic membranes [46]. Immunohistochemistry revealed that in the CNS, Cend1 is widely expressed throughout the gray matter of the brain and spinal cord. In the peripheral nervous system (PNS), Cend1 is expressed in the perikarya and processes of dorsal root ganglion neurons, as well as in the myelinated and unmyelinated neuronal processes of the dorsal roots. Cend1 was also detected in neuronal cell bodies and fibers of the enteric ganglia. In addition, Cend1 expression was observed in primary cultures of neurons derived from pig and rat brains [19, 46, 47]. Notably, Cend1 expression is first detectable at embryonic day (E) 12.5 in the rat brain and is increased in an age-dependent manner, reaching a maximum at postnatal day (P) 15 [20].

Although Cend1 was identified as a protein expressed in postmitotic neurons [19–21, 46], subsequent studies revealed that it marks neural stem/progenitor cells all along the different stages of the neuronal lineage as well as terminally differentiated neurons, both in rodents and in chicks [15–18]. Cend1 is expressed at low levels in neuronal progenitors, including radial glia, while its expression is distinctly upregulated in young postmitotic neurons and remains high in mature neurons [15, 20, 21]. Especially in the embryonic rodent forebrain, Cend1 is expressed in multipotential progenitor cells before terminal mitosis and in their neuronal progeny during the neurogenic period [15]. Double-labeling experiments with 5′-bromo-2′-deoxyuridine (BrdU) and ^3^H-thymidine to define a cohort of proliferative progenitors that exit S phase in synchrony, revealed that Cend1 is associated with the dynamics of asymmetric/neuron-generating divisions [15].

### 3.2. Adult Neurogenesis

Over the last two decades, it has become apparent that persistent neurogenesis throughout life occurs in two specific brain areas of the adult mammalian forebrain: the subventricular zone (SVZ) adjacent to the lateral wall of the lateral ventricles and the subgranular zone (SGZ) of the hippocampal dentate gyrus (DG) [48–55]. In the adult SVZ, a subset of relatively quiescent GFAP^+^ radial cells (type B cells) has the potential to serve as adult neural stem cells and generate rapidly dividing, transit-amplifying nonradial progenitors (type C cells), which in turn give rise to neuroblasts (type A cells) that migrate through the rostral migratory stream (RMS) to the olfactory bulb (OB). In the adult SGZ, a population of GFAP^+^/Sox2^+^ radial cells corresponds to quiescent neural stem cells (type 1 cells). They coexist with actively proliferating, GFAP^+^/Sox2^+^ nonradial NPCs (type 2 cells) that generate both astrocytes and neuroblasts. Neuroblasts then migrate into the granule cell layer and mature into neurons [54]. This process is sustained by the life-long persistence of neural stem cells in these two areas, with the SVZ being the richest source of neural stem/progenitor cells in the adult brain, thus providing a continuous supply of newborn neurons to the olfactory bulb (OB). Cend1 is expressed in the adult SVZ, while its expression rises along the adult mouse SVZ-RMS-OB pathway [15, 22]. In particular, Cend1 is expressed in type B and transit-amplifying type C progenitor cells of the adult SVZ and its expression increases concomitantly with the degree of neuroblast maturation. In addition, Cend1 is highly expressed by NeuN^+^ and GABA^+^ interneurons of the OB [22]. Taken together, these data indicate that Cend1 is present in neuronal progenitors and postmitotic neurons along the SVZ-RMS-OB pathway, thus marking the neuronal lineage in this region of the adult brain.

## 4. Cend1 Expression in the Developing Chick and Mouse Spinal Cord Is Inversely Correlated with the Proliferation Gradient

Early in development, a population of ectodermal cells is committed to neural fate, to form the neural plate and later to produce the neural tube, of which the anterior part generates the forebrain and the posterior part the spinal cord. The spinal cord develops from a small number of highly plastic neural stem/progenitor cells that proliferate, acquire regional identities, and generate a progressively restricted repertoire of cell types, first neurons and later glial cells, oligodendrocytes, and astrocytes. Soon after the onset of neural induction, the anteroposterior (rostro-caudal) identity of progenitors is specified and the hindbrain and spinal cord adopt their posterior identity. This is achieved by signaling centers located in the neural tube and surrounding tissues that produce fibroblast growth factors (FGFs), bone morphogenetic proteins (BMPs), retinoids, and Wnt proteins [1, 56–59]. The result of this early patterning is a structure in which distinct anteroposterior segments are defined by the expression of combinations of different transcription factors, mainly members of the Hox family [59, 60]. Similarly, after neural tube formation, dorsoventral organization is already acquired. The proliferation rate of neural progenitors is higher in the dorsal as compared to the ventral part, whereas the opposite is true for the differentiation rate [18, 61]. In this early neural tube, the region between the roof plate (RP) and floor plate (FP) is densely occupied by neural progenitors. The identity of these progenitors is modulated along the dorsoventral axis by extrinsic signals that activate hierarchies of transcription factors, expressed in a region- and cell-specific manner, to regionalize the early neural tube [1, 2, 18]. Floor plate differentiation is mediated by inductive signaling from the notochord and especially by the secreted protein Sonic hedgehog (Shh). Shh expression gradient is increased dorsoventrally and controls ventral patterning by the repression of the homeodomain proteins Pax6, Pax7, Dbx1, Dbx2, and Irx3 and the induction of expression of Nkx6.1 and Nkx2.2 homeodomain transcription factors [1].

Expression studies in the developing chick and mouse spinal cord confirmed the expression of Cend1 by cycling neuroepithelial precursors located in the ventricular zone (VZ) and by postmitotic neurons of the mantle zone (MZ). Cend1 mRNA expression was first detected in the MZ of chick spinal cord at HH stage 15 embryos. Less intense Cend1 expression was observed in the proliferative VZ at the same embryonic stage [16–18]. In accordance, Cend1 expression is detected at E12.5 mouse embryo in the spinal cord and brain, at a time when neurogenesis takes place throughout the rostro-caudal axis of the CNS [17].

Interestingly, in the embryonic mouse and chick spinal cord, a mediolateral and dorsoventral gradient of Cend1 expression is apparent, with lower Cend1 levels in the neural stem/progenitor cell population of the VZ and higher in the differentiated cells of the MZ in the mediolateral axis. In the dorsoventral axis, highest Cend1 expression levels are evident in ventral and lowest in dorsal areas [17, 18]. These expression gradients are, on one hand, inversely correlated with the proliferation gradients and, on the other hand, proportionate to the differentiation gradients existing in the spinal cord, where proliferation is higher in dorsal and medial areas, in contrast to differentiation which persists in ventral and lateral areas [18, 61], suggesting that Cend1 may be functionally involved in these processes [18].

## 5. Functional and Mechanistic Studies in Neuro2a Cells

### 5.1. Cend1 Promotes Cell Cycle Exit through the p53/Cyclin D1/pRb Signaling Pathway

Functional evidence supporting the dual role of Cend1 in coordination of cell cycle exit and neuronal differentiation came from analysis of its overexpression in the mouse neuroblastoma Neuro2A cell line, which is inherently capable of neuronal differentiation. Stably transfected Neuro2A cells overexpressing Cend1 exhibited a significant change in morphology, reflected by enhanced process outgrowth and a slower rate of division [26]. Moreover, in the presence of retinoic acid, these cells exhibited an accelerated morphological and molecular differentiation. Cend1 could also induce accelerated neuronal differentiation of mouse embryonic teratocarcinoma P19 cells upon treatment with retinoic acid [62] with concomitant downregulation of the pluripotency marker Oct3 and upregulation of the proneural genes Neurogenin 1 and Mash1 [63].

**Figure 2:**
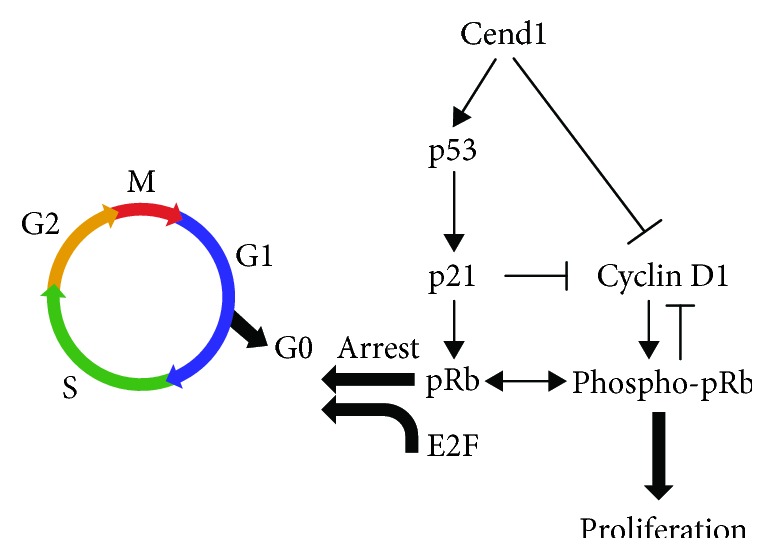
Cend1 induces the p53-Cyclin D1-pRb signaling pathway resulting in cell cycle arrest at the G1/G0 phase of the cell cycle. Cend1 triggers p53 and its downstream effector p21, leading to pRb hypophosphorylation and withdrawal from the cell cycle at the G1/G0 transition, while interfering with Cyclin D1 signaling.

Gain and loss-of-function approaches in Neuro2A cells by means of Cend1 overexpression or knockdown provided direct evidence that Cend1 activates the p53-pRb signaling pathway controlling the balance between cell proliferation and cell cycle exit [23]. Moreover, the antiproliferative effect of Cend1 is associated with Cyclin D1 downregulation and its cytoplasmic relocation from the nucleus, concomitantly to pRb hypophosphorylation resulting in withdrawal from the cell cycle at the G1/G0 transition point [23] (Figure 2).

Interestingly, overexpression of Cend1 in 3T3 fibroblasts triggered cell cycle exit, but apparently, because of absence of the appropriate cellular machinery required for neuronal differentiation, these cells were driven towards a proapoptotic pathway, suggesting a context-dependent cellular function for Cend1 [23]. As the antiproliferative effect of Cend1 could be extended to nonneural cells, coupled to proapoptotic induction, we considered the possibility that Cend1 has a more generalized antitumor action that may become of use in cancer therapeutics. Histone deacetylase inhibitors (HDACIs) have been reported to arrest cell cycle and induce differentiation [64, 65]. Especially, trichostatin A (TSA), which is a potent inhibitor of histone deacetylases [65], inhibits growth and induces differentiation and apoptosis in Neuro2A cells [66–68]. In this context, it was demonstrated that TSA stimulates indirectly Cend1 promoter activity and upregulates Cend1 mRNA and protein levels in Neuro2A cells. The operation of such an indirect mechanism for regulating the transcriptional repression/activation of Cend1 in neuronal cells is further supported by the fact that HDAC inhibition is not sufficient to derepress Cend1 promoter activity in nonneural cells [69]. TSA-mediated upregulation of Cend1 is associated with a concomitant p53-independent induction of p21 [69, 70], suggesting that Cend1 may participate in both p53-dependent [23] and independent pathways leading to cell cycle arrest [24, 69], as we will discuss below.

### 5.2. Cend1, RanBPM, and Dyrk1B Tripartite Interactions in Cell Cycle Progression/Exit

Although the mechanistic studies described above elucidated Cend1-associated antiproliferative pathways, the protein partners interacting directly with Cend1 remained elusive. Using a yeast two-hybrid system Ran-binding protein M (RanBPM) was identified as a direct interacting partner for Cend1 via its SPRY-LISH-CTLH domain [24]. RanBPM is a multidomain intracellular protein that shuttles between the cytoplasm and the nucleus and has been shown to act as a scaffold for signal transduction for several receptors, nuclear proteins, transcription factors, and cytoplasmic kinases in the immune and nervous systems [71]. Among its different functions, RanBPM has been implicated in cell cycle progression of neuronal precursors via an as yet unknown mechanism [72], while it has been identified as a binding partner for the growth arrest protein Dyrk1B in lung epithelial cells [73]. Dyrk1B belongs to the nuclear family of dual-specificity tyrosine phosphorylation-regulated kinases (Dyrks), which include several vertebrate, invertebrate, and lower eukaryotic orthologs characterized by highly conserved Dyrk homology and kinase domains [74, 75]. Mammalian Dyrk1A and Dyrk1B and the *Drosophila* minibrain kinases have been shown to affect proliferation and/or differentiation in a variety of cell types [74–77]. Moreover, Dyrk1B has been reported to control Cyclin D1 levels by promoting Cyclin D1 phosphorylation and its subsequent degradation [78–80]. These observations triggered the hypothesis for a tripartite interaction between Cend1, RanBPM, and Dyrk1B in neuronal cells, which was further investigated.

**Figure 3:**
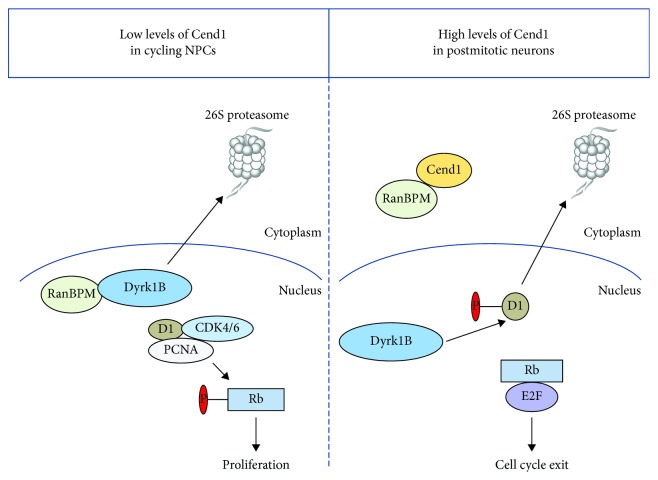
Model of tripartite protein-protein interactions between Cend1, RanBPM, and Dyrk1B regulating NPC cell cycle progression/exit and neuronal differentiation. In NPCs, where expression of Cend1 is low, RanBPM binds Dyrk1B in the nucleus and facilitates its degradation by the 26S proteasome. Thus, Cyclin D1 remains active in the nucleus to drive cell cycle progression. Before terminal mitosis of NPCs, Cend1 expression rises and stays high in postmitotic neurons where it binds to and restrains RanBPM to the cytoplasm. Consequently, Dyrk1B kinase is maintained intact in the nucleus, where it signals cell cycle exit by phosphorylation of Cyclin D1 resulting in its degradation.

Cend1, RanBPM, and Dyrk1B are expressed in the mouse brain and in cultured embryonic cortical neurons, while RanBPM can form separate complexes with either of the two other proteins when expressed in HEK293T cells [24]. Further, in Neuro2A cells, a Cend1-dependent or Dyrk1B-dependent downregulation of Cyclin D1 was observed that could be reversed following their interaction with RanBPM. Interestingly, binding of RanBPM to either Cend1 or Dyrk1B stabilized Cyclin D1 in the nucleus and increased 5-bromo-2′-deoxyuridine (BrdU) incorporation, which was used as a measure of cellular proliferation [24]. In the case of Dyrk1B-RanBPM interaction, this occurred because RanBPM facilitated Dyrk1B proteasomal turnover. However, when all three proteins were coexpressed, Dyrk1B was rescued in the nucleus to target Cyclin D1. Additionally, coexpression of RanBPM with either Cend1 or Dyrk1B also had a negative effect on Neuro2A cell differentiation in the presence of retinoic acid, as compared with cells expressing each protein separately. These results demonstrated that functional interactions between Cend1, RanBPM, and Dyrk1B influence the balance between cellular proliferation and differentiation in Neuro2A cells, suggesting that the three proteins may also play a similar role in cell cycle progression/exit and differentiation of neuronal precursors during neurogenesis [24]. This is in agreement with the fact that both RanBPM and DyrK1B are expressed in neuronal precursors in parallel with Cend1 [72] (Kokkorakis, Matsas, and Gaitanou unpublished data). A model of Cend1, RanBPM, and Dyrk1B interactions in NPCs and neurons is presented in Figure 3. In NPCs, where expression of Cend1 is low, RanBPM interacts with Dyrk1B promoting its degradation by the 26S proteasome. Thus, Cyclin D1 remains active in the nucleus to drive cell cycle progression by association with the CDK4/6-PCNA and phosphorylation of the retinoblastoma (Rb) protein. Before terminal mitosis of NPCs, Cend1 is elevated and remains high in postmitotic neurons, where Cend1 binds to RanBPM in the cytoplasm. Consequently, Dyrk1B kinase is maintained intact in the nucleus to phosphorylate Cyclin D1 and induce its degradation by the 26S proteasome, resulting in the cell cycle exit (Figure 3). To date, our findings suggest two mechanisms by which Cend1 controls Cyclin D1 levels and promotes cell cycle exit in neuronal precursors during neurogenesis.

Considering its interaction with RanBPM, it is possible that Cend1 might also play a role in mitotic regulation. RanBPM has been previously implicated in the progression of neuronal precursors through the M phase at the surface of the neocortical ventricular zone [72]. This is an interesting observation, given the implication of Cend1 in the transition from a symmetric to an asymmetric pattern of cell divisions [15]. Since this transition is an important determinant of neuronal production during brain development, it would be important to investigate further the role of Cend1 in this process. Interestingly, using GST pull-down assays combined with proteomics profiling, we were recently able to show that Cend1 interacts with the cytoskeletal dihydropyrimidinase-related protein-2 also known as Collapsin response mediator protein 2 (CRMP2) (Gaitanou and Matsas, unpublished observations). CRMP2 is a microtubule-associated protein that plays a critical role in dividing cells. It stabilizes the mitotic apparatus during cell division while CRMP2-depleted cells exhibit destabilized anaphase, reduced astral microtubules, and altered spindle position [81]. Investigation of the consequences of Cend1-CRMP2 interaction in mitosis is in progress and could well explain how Cend1 affects the transition from symmetric to asymmetric/neuron-generating cell divisions [15].

### 5.3. Cend1 Promotes Neuronal Differentiation by Suppressing Notch Signaling

The most important observations concerning the neurogenic activity of Cend1 have come from studies in the developing spinal cord. Cend1 is sufficient to initiate the differentiation of spinal cord neural precursors toward acquisition of generic neuronal- and subtype-specific traits. Gain-of-function approaches in the neural tube of the chicken embryo, where Cend1 forced expression was promoted by unilateral electroporation, showed that Cend1 negatively regulates proliferation of neuronal precursors, driving them to prematurely exit the cell cycle, downregulate Notch1, Hes5, Olig2, Cash1, and Pax7, and commit to a neuronal differentiation pathway [16]. Conversely, loss-of-function conferred by siRNA targeting Cend1 in neural progenitor cells (NPCs) derived from E12.5 mouse spinal cord enhanced proliferation and impaired neuronal differentiation, further corroborating that Cend1 participates in the molecular machinery that coordinates cell cycle exit and differentiation of neuronal precursors [16]. The combined effect on proliferation and differentiation results in precocious induction of neurogenesis and generation of postmitotic neurons within the ventricular zone. The dual function of Cend1 is not recapitulated by overexpression of the cell cycle inhibitor p27Kip1, suggesting that cell cycle exit does not induce differentiation by default. Thus, Cend1 is capable not only to cause exit of neuronal progenitors from the cell cycle but also to coordinately induce neuronal differentiation. Moreover, induction of endogenous Cend1 is promoted by forced expression of the proneural gene Mash1, indicating that Cend1 is a part of the differentiation program activated and regulated by proneural genes [16].

Suppression of Notch1 signaling by Cend1 may be mediated through the Dyrk family of proteins [76]. Considering the negative correlation between the functions of Notch and Cend1, it seems a reasonable assumption that Cend1 might be the target of basic helix-loop-helix proneural genes, thus defining a late molecular switch for generic neurogenesis [16]. Because proneural genes are expressed transiently in neural progenitors and are usually downregulated before progenitor cells exit the VZ and begin to differentiate [16, 82–84], their ability to potentiate full neuronal differentiation relies on the induction of downstream differentiation genes that can further implement neuronal differentiation programs [3, 16]. Cend1 is a strong candidate for such a function, whereas induction of endogenous Cend1 after misexpression of Mash1 in the chick neural tube strongly supports this hypothesis. In agreement, Neurogenins 1 and 2 directly transactivate the human Cend1 promoter [22, 85].

## 6. Cend1 as a Valuable Gene Therapy Tool

### 6.1. Cend1 Can Be Used as a Therapeutic Gene for Brain Repair

The adult cerebral cortex has limited ability for regeneration after brain damage, due to the lack of a resident population of neural stem/progenitor cells responsive to injury-induced signals. Limited compensatory cortical neurogenesis has been reported following stroke [78] or induced apoptotic degeneration [86], but the number of neurons produced is insufficient to replenish neuronal loss after injury and restore cortical function [87, 88]. To overcome this limitation, efforts have been made to stimulate the endogenous NPC population of the neighboring subventricular zone (SVZ) with growth factors, in order to recruit a population of NPCs to the lesioned cortex. Moreover, transplantation of suitable cell types has also attracted considerable interest as an alternative strategy to overcome the regenerative limitations of the lesioned brain [89–91]. NPC transplantation has been combined with ex vivo gene delivery for expression of regeneration-promoting molecules. So far, several studies have addressed the efficacy of transplanting embryonic stem cells preconditioned to restrict their differentiation to neural lineages as well as neural stem/precursor cells from the embryonic or adult CNS into animal models of brain and spinal cord injury [92–94]. Additionally, a number of molecules have been used to enhance the capacity of embryonic stem cells and NPCs to repair CNS damage in animal models of brain injury. These include trophic factors such as NGF [95], GDNF [96], EGF [97], FGF2 [98], IGF1 [99–101], and cytokines such as erythropoietin [102] and also cell adhesion molecules [103–106] and extracellular matrix proteins [107].

Due to its neurogenic activity, Cend1 is a promising candidate for ex vivo gene therapy approaches aiming at neuronal replacement. Towards this direction, genetically modified NPCs from E14.5 mouse brain overexpressing Cend1 with the help of a lentiviral vector were grafted in a mouse model of acute cortical injury [108]. Extensive cellular loss of NeuN^+^ neurons was observed the day following the injury while GFAP^+^-reactive astrocytes started to populate the area around the lesion site, indicating an early response to injury. Four weeks postinjury, GFAP^+^-reactive astrocytes formed a glial scar that surrounded and limited the injury site while transplanted cells were found within the lesion. Cend1 overexpression enhanced the differentiation of the grafted NPCs into neurons, which acquired a GABAergic interneuron phenotype [108]. An interesting, potentially beneficial effect was the significant reduction of astrogliosis in animals that had received NPCs with Cend1 overexpression. This was demonstrated by a decrease in the density of activated astrocytes present within the glial scar, as well as the degree of hypertrophy of the activated astroglial cells. It is noteworthy that the effect of Cend1 was specifically targeted to the GFAP^+^ cells forming the gliotic scar, whereas no effect was noted on the host NG2^+^ glial precursors participating in glial scar formation, which are thought to contribute to tissue regeneration [108, 109]. Cend1 effect on astrogliosis suggested for the first time a noncell autonomous function, which probably impacts on Cyclin D1-dependent regulation of astrocyte proliferation and possibly also on their survival, deserving further study. Cend1 involvement in regulation of stimulus-induced intracellular calcium mobilization may provide a link to explain the propagation of Cend1 action to the host cortical tissue [110].

Glial scar formation around the injured area has both beneficial and detrimental consequences after CNS insults. Initially, it is fundamental for sealing off the injured tissue and restricting inflammation and neuronal death [111, 112], but at later stages, it inhibits regeneration [113, 114]. Cend1-overexpressing grafts were found to attenuate astrogliosis one month after injury when the adverse effects of activated astroglia are likely to outnumber their positive contributions, without impinging on lesion size [108]. As such, the reduction in astrogliosis by Cend1 overexpression should contribute in enhancing regenerative processes leading to repair. Therefore, the enhanced neuronal output within the graft and the attenuation of astrogliosis render Cend1 a promising candidate for cell replacement approaches, especially if used in combination with other regenerative molecules.

### 6.2. Cend1 Minimal Promoter, a Gene Therapy Tool for Neuron-Specific Gene Delivery

The human *Cend1* gene maps to chromosome 11p15.5, a region characterized by genomic imprinting, an epigenetic phenomenon that causes genes to be expressed in a parent-of-origin-specific manner [21]. This chromosomal region is implicated in Beckwith-Wiedemann syndrome (BWS), an overgrowth genetic disorder, as well as in several types of embryonic, childhood, and adult cancers [115–119]. The human *Cend1* gene contains a promoter with multiple transcription start sites, lying within CpG islands and lacking TATA boxes. Within the promoter region, there are four functional Sp1-binding sites. Simultaneous mutations of all four Sp1 sites resulted in complete loss of promoter activity. Transactivation experiments revealed that Sp1 directly activates the Cend1 promoter, while activation also occurs in the presence of Neurogenin 1 [85]. Functional studies of the human *Cend1* promoter in neural and nonneural cell lines revealed that it is preferentially active only in neural cells. Moreover, deletion analysis revealed a minimal promoter fragment of 88 bp, which is sufficient to drive and restrict reporter gene expression in primary neurons, but not in glial cells [85].

Neurogenins 1/2 and Olf-1 binding sites are both present on the minimal human *Cend1* promoter. Neurogenins 1/2 and Olf-1 act upstream of NeuroD to promote neurogenesis [120–122]. Interestingly, Neurogenins 1/2 directly transactivate the human Cend1 promoter in the ND26 neuronal cell line, as well as in neurospheres [22, 85], confirming that Cend1 is part of the differentiation program activated by proneural genes during neurogenesis. Importantly, alleviation of Cend1 activation by Neurogenin 1 upon mutation of the E-box consensus sequence present in the Cend1 proximal promoter that constitutes a putative bHLH protein binding site [123] indicates a direct action of Neurogenin 1 on Cend1 transcription [22, 85]. Since proneural genes are transiently expressed in precursor cells and are readily downregulated in differentiated neurons, it appears that their ability to sustain neuronal differentiation relies on activation of downstream genes, including Cend1, participating in cellular differentiation networks.

The short but potent Cend1 promoter fragment of 88 bp may be useful for the development of novel, neuron-specific gene therapy tools. Such an approach has been recently developed through generation of adeno-associated viral vector serotype 8 (AAV8) for neuron-specific delivery of therapeutic genes in the CNS [124]. AAV8 vectors carrying enhanced green fluorescent protein as reporter gene under the transcriptional control of five different small-sized CNS-specific promoters were made. Specifically, three glia-specific vectors were constructed using two truncated forms of the human promoter for glial fibrillar acidic protein (GFAP) as well as a truncated form of the murine GFAP promoter. All three resulted in primarily glial expression *in vivo* after stereotactic injection in the mouse brain. On the other hand, robust neuron-specific expression was observed using the minimal promoters for the neuronal protein Cend1 and the neuronal nicotinic receptor *β*2 (CHRNB2). The Cend1 minimal promoter conferred the strongest GFP expression to a high percentage of transduced neurons [124], indicating that it is a valuable tool for gene therapy approaches.

## 7. Cend1 Is Necessary for Formation of a Structurally and Functionally Normal Brain: Lessons from Cend1 Knockout Mice

To gain further insight into the physiological function of Cend1, we generated knockout mice which completely lack Cend1 [125]. Homozygous Cend1^−/−^ mice are viable and fertile with a life expectancy that does not differ from that of wild-type animals. Although macroscopically Cend1^−/−^ mice show no overt morphological defects in the brain, closer observations revealed a number of structural and functional deficits in the cerebellum and other brain regions, which are described below.

### 7.1. Impaired Cerebellar Development and Deficits in Motor Coordination in Mice Lacking Cend1

The cerebellum is a primary site for motor coordination, and lesions in this region result in ataxia, a movement incoordination disorder. Two types of neurons present in the cerebellar cortex play dominant roles: the Purkinje cells and the granule cells [126, 127]. Granule cell precursors (GCPs) generated in the embryonic rhombic lip move across the developing cerebellar surface to form the external granule layer (EGL). GCPs in the outer part of the EGL proliferate extensively during the first postnatal week and then migrate inwards, through the Purkinje cell layer to form the internal granule layer (IGL) [128, 129]. GC radial migration is contact guided by Bergmann glial cell processes [130]. Purkinje cells from the germinal zone of the fourth ventricle migrate towards the cerebellar surface and settle into a monolayer where postnatally, they differentiate and develop an extensively arborized dendritic tree [131]. Purkinje cell dendrites are targeted by GC axons, the parallel fibers, and together constitute the bulk of the molecular layer (ML). Cerebellar development is complete after the third postnatal week in mice.

Genetic ablation of Cend1 leads to irregularities in cerebellar layering arising from increased proliferation of GCPs, delayed radial granule cell migration, and impaired Purkinje cell differentiation, leading to ataxic gait and deficits in motor coordination. In particular, GCPs of P0–P9 Cend1^−/−^ mice show increased proliferation and significant Cyclin D1 upregulation, resulting in expansion of the EGL. Cyclin D1 expression is prominent in GCPs of the EGL [132], which proliferate under the influence of the potent mitogen Sonic hedgehog (Shh) secreted by Purkinje cells [133]. Cyclin D1 is a direct target of Shh signaling [134] and is mediated by its receptor Patched1. Patched1 is expressed in GCPs, and upon binding of Shh, the inhibition of smoothened-mediated signal transduction is alleviated thus triggering activation of the Gli family of transcriptional regulators, which in turn promote downstream proliferation events [135]. In relevance, altered levels of Patched1 are found in the cerebellum of Cend1^−/−^ mice confirming aberrant Shh signaling [125].

Delayed radial GC migration was also observed in Cend1^−/−^ mice cerebella, as a consequence of reduced BDNF expression. Nevertheless, despite their increased proliferation and delayed migration, GC differentiation and extension of parallel fibers were not affected in Cend1^−/−^ mutant mice [125].

By contrast, Cend1^−/−^ mice exhibit an obvious disruption in Purkinje cell differentiation. A largely stunted dendritic arborization was evident in the Purkinje cells of early postnatal mutant animals that was maintained in adulthood. Purkinje cells elaborate a complex and highly branched dendritic arbor, which allows them to integrate numerous signals from the cerebellar circuitry. The stunted branching of Purkinje cell dendrites observed in Cend1^−/−^ mice is a phenotype also seen upon Reelin deficiency [136]. Interestingly, Reelin has been implicated in dendrite maturation not only in cerebellar Purkinje cells but also in hippocampal neurons [137]. Strikingly, the expression of Reelin was reduced by 30–40% of normal levels in mice lacking Cend1. Reelin is secreted by cells in the EGL as well as in the deep nuclei of the developing cerebellum and is a major player in the migration and positioning of Purkinje cells in a monolayer, a process completed by the first postnatal week [138, 139]. Thus, in the homozygous *reeler* mutant mouse which contains a mutation in the gene-encoding Reelin, 95% of Purkinje cells are ectopic [140]. However, in the Math1-null mutant mice, which completely lack EGL cells, the majority of Purkinje neurons migrate normally [141], suggesting that residual Reelin secreted from the deep cerebellar nuclei is sufficient for proper positioning of these cells [142], thus explaining the fact that normal Purkinje cell migration is observed in Cend1 mutant mice.

**Figure 4:**
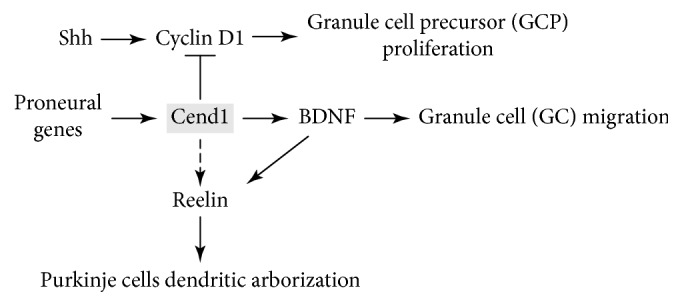
Cend1-associated pathways during cerebellar development. Cend1 negatively regulates proliferation of granule cell precursors (GCPs) via inhibition of Shh/Cyclin D1. In addition, Cend1 promotes BDNF secretion which induces Reelin-dependent GC migration and Purkinje cell differentiation.

Heterozygous *reeler* mice do not have an overt phenotype but manifest perturbed dendritic differentiation of Purkinje cells [143] in line with our observations in Cend1 mutants. Reelin exerts its function by binding to and activating the very low-density lipoprotein receptor and the apolipoprotein E receptor 2 [136]. Both receptors bind mDab1 on their cytoplasmic tails, an adaptor protein related to the Drosophila-disabled gene product. Targeted disruption of components of this pathway either in knockout mice or in the naturally occurring *reeler* and *scrambler* mutant mice results in similar neuropathological features [144]. These include abnormal dendritic arborization of Purkinje cells as in Cend1 mutants. These findings suggest that Cend1 influences directly or indirectly the Reelin signaling pathway [125] (Figure 4). As expected, abnormal cerebellar layering and morphological defects in Purkinje cells in Cend1^−/−^ mice are associated with locomotion and learning deficits [125].

Interestingly, a study that produced an interaction network for 54 proteins involved in 23 human inherited ataxias characterized by loss of balance due to cerebellar Purkinje cell (PC) degeneration revealed that Cend1 is a direct protein partner of ataxin-1-interacting protein (A1Up) [145]. This interactome map provides a tool for better understanding of pathogenic mechanisms common for this class of neurodegenerative disorders and for identifying candidate genes for inherited ataxias, providing an additional link for the role of Cend1 in cerebellar development and function.

Another intriguing finding that implicates Cend1 in human disease came from a study showing that Cend1 associates with Ahi1 [31], also known as Jouberin [146], a protein encoded by the Abelson helper integration-1 (AHI1) gene. AHI1 mutations lead to Joubert syndrome, a rare autosomal recessive disorder characterized by an abnormal brain structure, cerebellar hypoplasia, retinal dystrophy, renal degeneration, and delayed development [147–154].

### 7.2. Cend1 Expression Is Necessary for Generation of the Right Numbers of GABAergic Neurons in the Cortex, Hippocampus, and Amygdala

Further studies in Cend1^−/−^ mice revealed functional deficits in a range of behaviors, including anxiety and exploratory behavior in the elevated plus maze and open field, associative learning in fear conditioning, and spatial learning and memory in the Morris water maze [155]. These observations were associated with reduced numbers of GABAergic interneurons, but not glutamatergic neurons, in functionally relevant brain areas, including the cortex, amygdala, and hippocampus. Association of Cend1 with a GABAergic fate is in agreement with previous observations demonstrating that Cend1-overexpressing NPCs adopt a GABAergic phenotype after transplantation in the lesioned cerebral cortex [108] as well as with the fact that Cend1 drives reprogramming of cortical astrocytes to a GABAergic neuronal fate when used as a cellular reprogramming factor [156].

GABAergic interneurons comprise approximately one-fifth of the total neuronal population in the adult cortex and play central roles in cortical circuitry and activity. They provide the main source of inhibition to cortical circuits and regulate the activity of excitatory projection neurons [157, 158]. Previous studies have indicated that alterations in the number, function, and distribution of cortical interneurons are associated with a variety of severe neurological disorders such as schizophrenia, autism, and epilepsy [159, 160]. Cortical interneurons originate from the ganglionic eminences (GEs), mostly the medial ganglionic eminence (MGE), which is a well-defined domain of the subpallial ventricular area, and migrate tangentially to populate the developing cortex [161, 162]. Apart from cortical interneurons, the GE gives rise to GABAergic interneuron subtypes that contribute to the mammalian amygdala [163]. This region is subdivided to the lateral, basolateral (BLA), and central amygdala and has been identified as a key anatomic structure of the circuitry that mediates fear conditioning [164, 165]. An important function of this region is to control behaviors that are related to fear and anxiety, in concert with the cerebral cortex and the hippocampus [166] whose GABAergic interneurons also originate from the GEs [167].

In agreement with Cend1 function, the reduced numbers of GABAergic interneurons in the adult Cend1^−/−^ cortex and amygdala correlated with increased proliferation and apoptosis as well as with reduced migration of neuronal progenitors from the embryonic GE from which these cells originate. Additionally, we noted aberrant neurogenesis in the adult dentate gyrus of the hippocampus, which is a key structure in learning and memory [155]. In this region, new neurons are produced throughout adult life and become functionally integrated in preexisting neuronal circuits [52, 168–172]. An increasing number of studies indicate a functional link between hippocampal-dependent learning and adult hippocampal neurogenesis [53, 173–177]. Our analysis showed an activation of the earlier stages of adult hippocampal neurogenesis in Cend1^−/−^ mice, accompanied by increased cell apoptosis. These events may be causally related to a parallel decrease in local Parvalbumin interneurons (a subtype of GABAergic cells), which are known on one hand to suppress the activation of neural stem cells [171] and support the survival of newborn neurons on the other [178]. Taken together, our data highlight the requirement for Cend1 expression in the formation of a structurally and functionally normal phenotype.

## 8. Cend1 in Zika Virus Infection

In an important recent study aiming to understand how Zika virus (ZIKV) affects neuronal cells, Scaturro and colleagues applied an integrated approach in human NPCs and the neuronal cell line SK-N-BE2 to characterize cellular responses to viral infection at the proteome and phosphoproteome levels [25]. Further, they used affinity proteomics to identify cellular targets of ZIKV proteins, using as baits three structural and seven nonstructural viral proteins (NS1, NS2A, NS2B, NS3, NS4A, NS4B, and NS5). These 386 ZIKV-interacting proteins were identified possessing ZIKV-specific or pan-flaviviral activities, as well as host-interacting proteins with known functions in neuronal development, retinal defects, and infertility. Among these, Cend1 was found to interact specifically with the nonstructural viral protein NS4B, which has been previously implicated in inhibition of neuronal development [179]. The NS4B-Cend1 interaction was validated upon ZIKV infection of SK-N-BE2 cells transduced with a lentivirus expressing Cend1, followed by reciprocal coimmunoprecipitation experiments. Moreover, to assess the functional relevance of the newly identified host proteins, 17 selected cellular ZIKV partners including Cend1 were further evaluated for their implication in ZKV infection by gene silencing. Interestingly, Cend1 knockdown resulted in inhibition of ZIKV replication [25], suggesting that Cend1 is a promising new therapeutic target in Zika virus infection.

## 9. Concluding Remarks and Future Perspectives

The studies described herein have revealed that Cend1 is a protein expressed all along the neuronal lineage, from neural stem/progenitor cells to mature neurons. Cend1 plays a critical role in cell cycle progression/exit or apoptosis of neuronal progenitors that affect neuronal differentiation. Its combined actions thus contribute to the generation of a structurally and functionally normal brain. The search for Cend1-interacting partners, particularly in the developing vertebrate brain, could be further enriched with GST pull-down assays in the embryonic mouse brain homogenates combined with proteomics profiling or with proximity ligation assays. These approaches should help understand better the mechanism of action of this protein. Nevertheless, in line with its functional properties, Cend1 has been used successfully in direct reprogramming of mouse astrocytes to functional neurons [156], further highlighting its importance in acquisition of a neuronal fate. Moreover, in light of Cend1 antiproliferative activity in nonneural cells, which is coupled to proapoptotic induction, it is interesting to consider this molecule in cancer therapeutics. Finally, the recent finding that Cend1 is necessary for Zika virus infection opens up new perspectives for uncovering the multiple mechanisms that this virus has evolved to usurp, exploit, or perturb fundamental cellular processes, ultimately contributing to the broad spectrum of pathological abnormalities observed in humans. Future studies should delineate if Cend1 is a promising target for therapeutic interventions.
